# GRIPP2 reporting checklists: tools to improve reporting of patient and public involvement in research

**DOI:** 10.1186/s40900-017-0062-2

**Published:** 2017-08-02

**Authors:** S. Staniszewska, J. Brett, I. Simera, K. Seers, C. Mockford, S. Goodlad, D. G. Altman, D. Moher, R. Barber, S. Denegri, A. Entwistle, P. Littlejohns, C. Morris, R. Suleman, V. Thomas, C. Tysall

**Affiliations:** 1 Warwick Research in Nursing, Warwick Medical School, University of Warwick, Coventry, CV4 7AL UK; 2 Faculty of Health and Life Sciences, Oxford Brookes University, Oxford, UK; 3 Centre for Tropical Medicine and Global Health and UK EQUATOR Centre, University of Oxford, Oxford, UK; 4 Warwick Medical School, Coventry, UK; 5 Coventry University, Coventry, UK; 6 Centre for Statistics in Medicine, University of Oxford, Oxford, UK; 7 Centre for Journalology, Clinical Epidemiology Program, Ottawa Hospital Research Institute, Ottawa, Canada; 8 School of Health and Related Research, University of Sheffield, Sheffield, UK; 9 National Institute for Health Research, UCL School of Life and Medical Sciences, London, UK; 10 Kings College London, London, UK; 11 University of Exeter Medical School, Exeter, UK; 12 Public Involvement Programme, National Institute for Health and Care Excellence, London, UK

## Abstract

**Background:**

While the patient and public involvement (PPI) evidence base has expanded over the past decade, the quality of reporting within papers is often inconsistent, limiting our understanding of how it works, in what context, for whom, and why.

**Objective:**

To develop international consensus on the key items to report to enhance the quality, transparency, and consistency of the PPI evidence base. To collaboratively involve patients as research partners at all stages in the development of GRIPP2.

**Methods:**

The EQUATOR method for developing reporting guidelines was used. The original GRIPP (Guidance for Reporting Involvement of Patients and the Public) checklist was revised, based on updated systematic review evidence. A three round Delphi survey was used to develop consensus on items to be included in the guideline. A subsequent face-to-face meeting produced agreement on items not reaching consensus during the Delphi process.

**Results:**

One hundred forty-three participants agreed to participate in round one, with an 86% (123/143) response for round two and a 78% (112/143) response for round three. The Delphi survey identified the need for long form (LF) and short form (SF) versions. GRIPP2-LF includes 34 items on aims, definitions, concepts and theory, methods, stages and nature of involvement, context, capture or measurement of impact, outcomes, economic assessment, and reflections and is suitable for studies where the main focus is PPI. GRIPP2-SF includes five items on aims, methods, results, outcomes, and critical perspective and is suitable for studies where PPI is a secondary focus.

**Conclusions:**

GRIPP2-LF and GRIPP2-SF represent the first international evidence based, consensus informed guidance for reporting patient and public involvement in research. Both versions of GRIPP2 aim to improve the quality, transparency, and consistency of the international PPI evidence base, to ensure PPI practice is based on the best evidence. In order to encourage its wide dissemination this article is freely accessible on The BMJ and *Research Involvement and Engagement* journal websites.

**Electronic supplementary material:**

The online version of this article (doi:10.1186/s40900-017-0062-2) contains supplementary material, which is available to authorized users.

## Plain English summary

Patient and public involvement in health and social care research is increasingly important, helping to ensure that the research focuses on issues relevant to patients and the public. A wide variety of research papers with public involvement has been published over the past decade, yet many of these papers give little information about how members of the public were involved and what the result of this involvement was. This means that learning from these studies is limited. Working closely with patients and the public, we have developed guidance for people writing about public involvement to suggest what details to report. We carried out a thorough assessment of studies in this area and used a Delphi survey to ask 143 people who are knowledgeable about this topic for their opinions about what should be included in the guidance. The Delphi method consists of a series of questionnaires over a specific time period to find out whether there is agreement among experts about the topic under discussion. We found strong agreement on a number of issues to include in the guidance from the 112 people who completed three rounds of Delphi questionnaires. We also held a one day meeting to find out whether any additional issues for which we hadn’t reached agreement should be included in the guidance.

As a result of this three stage project, we developed two versions of the guidance, a short version of the guidance (GRIPP2-SF), which can be used when reporting public involvement in any study, and a long version (GRIPP2-LF) to use when the study is mainly about public involvement in research. Our aim in developing this guidance is to promote good quality reporting of public involvement, to inform good practice and create effective public involvement.

## Background

*The EQUATOR network has developed high standard* reporting guidelines such as the CONSORT (Consolidated standards of Reporting Trials) statement and the STROBE (Strengthening the Reporting of Observational Studies in Epidemiology) statement enhancing the quality of research reporting, but no guidance has been developed specifically for the reporting of patient and public involvement (PPI). This prompted the development of the original Guidance for Reporting Involvement of Patients and the Public (GRIPP), which tackled inconsistent reporting by helping researchers, patients, carers, and the public to improve the quality, consistency, and transparency of PPI reporting, to strengthen the quality of the international PPI evidence base [1]. While the original GRIPP checklist represented an important starting point in creating high quality PPI reporting, its development drew on systematic review evidence, without broader input from the international PPI research community [2–4]. Achieving consensus is now acknowledged as a crucial step in producing a reporting guideline [5]. GRIPP2 tackled this gap by developing consensus in the international PPI community.

INVOLVE defines public involvement in research as being carried out with or by members of the public rather than to, about, or for them. PPI in research can improve the relevance and overall quality of research, by ensuring that it focuses on the issues of importance to patients [1]. This includes, for example, working with research funders to prioritise research; the development of more patient relevant research questions, study designs, and outcomes; offering the patient perspective as members of a project steering group; commenting on and developing research materials to improve readability; assisting with recruitment to studies; lay write up of the studies; and advocacy of study results [2–4, 6–9]. In the UK, the National Institute for Health Research has provided vital strategic and infrastructure support to embed PPI across publicly funded research, creating a context where PPI is seen as a key element in research. Internationally PPI is also developing, with similar initiatives in Canada, United States, Australia, and Europe [10, 11]. Networks such as the citizen and patient involvement group of Health Technology International have evolved, enabling international collaboration in relation to involvement and engagement [12].

While the PPI evidence base has expanded significantly over the past decade, the reporting of PPI in papers has often been inconsistent and partial, with little information about the context, process, and impact of public involvement and with limited reporting of conceptualisation or theorisation [1–4]. Inadequate reporting can create problems for systematic reviews that attempt to synthesise PPI evidence [2–4]. Appraisal, interpretation, and synthesis of results are difficult, aside from the ethical imperatives of reporting research in a way that others understand and can use [13–16]. Inconsistent reporting creates a fragmented evidence base making it difficult to draw together our collective understanding of what works, for whom, why, and in what context. Furthermore, researchers, patients, carers, or clinicians cannot learn from previous experience, and precious resources devoted to involving patients and the public are wasted. Omitting descriptions of PPI activities from a study can represent a form of misreporting and might misrepresent the initial intentions of a study.

This article introduces the two versions of the GRIPP2 reporting checklist: GRIPP2-LF, a longer checklist for studies where PPI forms the primary focus of a study (Table 1) and GRIPP2-SF, a short checklist for studies where PPI is a secondary or tertiary focus (Table 2). We also describe the development of GRIPP2 and outline how it can be used.

**Table 1:** GRIPP2 long form

Section and topic	Item	Reported on page No
Section 1: Abstract of paper		
1a: Aim	Report the aim of the study	
1b: Methods	Describe the methods used by which patients and the public were involved	
1c: Results	Report the impacts and outcomes of PPI in the study	
1d:Conclusions	Summarise the main conclusions of the study	
1e: Keywords	Include PPI, “patient and public involvement,” or alternative terms as keywords	
Section 2: Background to paper		
2a: Definition	Report the definition of PPI used in the study and how it links to comparable studies	
2b: Theoretical underpinnings	Report the theoretical rationale and any theoretical influences relating to PPI in the study	
2c: Concepts and theory development	Report any conceptual or theoretical models, or influences, used in the study	
Section 3: Aims of paper		
3: Aim	Report the aim of the study	
Section 4: Methods of paper		
4a: Design	Provide a clear description of methods by which patients and the public were involved	
4b: People involved	Provide a description of patients, carers, and the public involved with the PPI activity in the study	
4c: Stages of involvement	Report on how PPI is used at different stages of the study	
4d: Level or nature of involvement	Report the level or nature of PPI used at various stages of the study	
Section 5: Capture or measurement of PPI impact		
5a: Qualitative evidence of impact	If applicable, report the methods used to qualitatively explore the impact of PPI in the study	
5b: Quantitative evidence of impact	If applicable, report the methods used to quantitatively measure or assess the impact of PPI	
5c: Robustness of measure	If applicable, report the rigour of the method used to capture or measure the impact of PPI	
Section 6: Economic assessment		
6: Economic assessment	If applicable, report the method used for an economic assessment of PPI	
Section 7: Study results		
7a: Outcomes of PPI	Report the results of PPI in the study, including both positive and negative outcomes	
7b: Impacts of PPI	Report the positive and negative impacts that PPI has had on the research, the individuals involved (including patients and researchers), and wider impacts	
7c: Context of PPI	Report the influence of any contextual factors that enabled or hindered the process or impact of PPI	
7d: Process of PPI	Report the influence of any process factors, that enabled or hindered the impact of PPI	
7ei: Theory development	Report any conceptual or theoretical development in PPI that have emerged	
7eii: Theory development	Report evaluation of theoretical models, if any	
7f: Measurement	If applicable, report all aspects of instrument development and testing (eg, validity, reliability, feasibility, acceptability, responsiveness, interpretability, appropriateness, precision)	
7 g: Economic assessment	Report any information on the costs or benefit of PPI	
Section 8: Discussion and conclusions		
8a: Outcomes	Comment on how PPI influenced the study overall. Describe positive and negative effects	
8b: Impacts	Comment on the different impacts of PPI identified in this study and how they contribute to new knowledge	
8c: Definition	Comment on the definition of PPI used (reported in the Background section) and whether or not you would suggest any changes	
8d: Theoretical underpinnings	Comment on any way your study adds to the theoretical development of PPI	
8e: Context	Comment on how context factors influenced PPI in the study	
8f: Process	Comment on how process factors influenced PPI in the study	
8 g: Measurement and capture of PPI impact	If applicable, comment on how well PPI impact was evaluated or measured in the study	
8 h: Economic assessment	If applicable, discuss any aspects of the economic cost or benefit of PPI, particularly any suggestions for future economic modelling.	
8i: Reflections/critical perspective	Comment critically on the study, reflecting on the things that went well and those that did not, so that others can learn from this study	

*PPI* patient and public involvement

**Table 2:** GRIPP2 short form

Section and topic	Item	Reported on page No
1: Aim	Report the aim of PPI in the study	
2: Methods	Provide a clear description of the methods used for PPI in the study	
3: Study results	Outcomes—Report the results of PPI in the study, including both positive and negative outcomes	
4: Discussion and conclusions	Outcomes—Comment on the extent to which PPI influenced the study overall. Describe positive and negative effects	
5: Reflections/critical perspective	Comment critically on the study, reflecting on the things that went well and those that did not, so others can learn from this experience	

*PPI* patient and public involvement

## GRIPP2 reporting checklist development methods

The study used the EQUATOR method for developing reporting guidelines [5], which included: systematic review evidence; a three stage Delphi survey including key stakeholders in the field of PPI; and a face-to-face collaborative meeting to develop consensus on items outstanding from the Delphi survey. A summary of methods is presented, with a companion paper reporting the rationale for GRIPP2 and the full methods [17]. For the purposes of this paper, we have therefore reported only a summary of key steps in Appendix 1.

The systematic reviews that underpinned the original GRIPP checklist had already identified the need for the guidance [2–4]. The PIRICOM systematic review, which included the conceptualisation, definition, measurement, impact, and outcomes of PPI on research, researchers, service users, participants, funders, and policy makers, was updated for GRIPP2 to ensure no additional concepts were omitted from the Delphi survey. In addition, searches were conducted to identify any other reporting guidelines for PPI.

Three rounds of the Delphi survey were conducted to gain consensus (see Appendix 2). This included 143 international participants in round one, with an 86% (123/143) response for round two and a 78% (112/143) response for round three reflecting the standard number of participants used in the development of previous EQUATOR guidance [5]. Participants of the Delphi survey included researchers, funders, patients, carers, editors, and individuals from international research agencies from countries including Australia, the United States, Canada, and Europe. Collectively, participants represented a wide range of expertise relevant to the development of consensus in PPI reporting.

Participants were asked to rate each item in the checklist on a scale of 1–10, with 1 considered unimportant and 10 considered very important, and medians and interquartile ranges were calculated for each item in the Delphi survey. Space next to each item was used for free text comments with suggested refinements, reiterations, and additional items. If items reached a median score of ≥8 in round one and round two they were considered to have reached positive consensus and included. Items that reached a median ≤ 5 in rounds one and two were excluded from the checklist. Items that reached a medium score of 6 or 7 in one round and a median score of ≥8 in the other round were voted on again in round three. Positive consensus was gained if the items scored a median score of ≥8 in two of the rounds. An important finding from the first round was that participants thought GRIPP items were most relevant when the main focus of a study was on PPI, and many felt there should be a shorter version for papers that included some element of PPI. As a result participants were asked to identify and score “core” items in round two which could be included in a shortened version of the guideline, suitable for studies that have included PPI as a secondary focus. The five core items that comprise the GRIPP2-SF all gained a median score of 9 in round two. Thus all five were included in round three and again gained median scores of 9, indicating that consensus was reached on the short form version.

Qualitative comments were analysed thematically to identify common themes, points of feedback, challenges to the items, and queries about wording [18]. Qualitative comments suggested the need to reword some items to simplify them and ensure clarity of meaning. Two sections from the original GRIPP checklist, section 5, which focused on measurement, and section 6, which was focused on capture of impact, were combined as it was recognised that they were conceptually overlapping. The original section 8 was deleted as participants thought it duplicated existing items.

Appendices 3 and 4 report the results of the Delphi survey. Following the Delphi survey, a collaborative consensus meeting was held with 25 key experts with knowledge, experience, or both, of PPI, including patient partners and carers (*n* = 8), researchers (*n* = 9), clinicians (*n* = 6), and healthcare journal editors (*n* = 2). The aim of this meeting was to finalise consensus on the seven items on the threshold of consensus following the Delphi survey (Appendix 2) and ensure clarity of the items.

Patient partners were collaboratively involved at key stages of the study. Three patient partners were recruited to the research team and were involved in refining the focus of the research questions, in development of the search strategy and interpretation of results of the systematic review, in discussions identifying the need for development of guidelines, and in selecting the items for the original GRIPP checklist. Furthermore, the patient partners assisted in developing the electronic survey for the first phase of the Delphi survey consensus process and were instrumental in assisting in recruitment to the Delphi study and in collation of comments from each Delphi survey round, and contributed to adapting items for GRIPP2. The consensus meeting involved eight patient partners in total, and the three patient partners recruited to the research team were involved in the write-up of the study and are coauthors in papers. More detailed information of their contribution to the development of GRIPP is described using GRIPP2-SF in Table 3 and used to populate the BMJ PPI guidance in Table 4.

**Table 3:** PPI in the development of GRIPP2 using GRIPP 2-SF^a^

Section and topic	Item
1: Aim Report the aim of the study	To develop international consensus on the key items to report to enhance the quality, transparency, and consistency of the PPI evidence base. To collaboratively involve patients as research partners at all stages in the development of GRIPP2
2: Methods Provide a clear description of the methods used for PPI in the study	Three patient partners were recruited to the research team to assist at all stages of the development of and consensus process for the GRIPP2 guidelines. They were involved in refining the focus of the research questions, in developing the search strategy, in interpreting results, in discussions identifying the need for development of guidelines, and in selecting the items for the original GRIPP checklist. The patient partners helped recruit participants (*n* = 60/143) to the Delphi survey through snowballing techniques. They helped pilot the electronic survey for the first phase of the Delphi survey consensus process and helped other patient reps with technical aspects of completing the online survey, hence improving the response rate in each round of the Delphi. They also worked with the researchers to collate comments from each Delphi survey round, to adapt items, and to feed back to the participants for the next Delphi survey round. They checked comprehension of changed items and comments from the lay perspective. The patient partners took part in the consensus workshop, alongside five other patients (*n* = 8/25 in total) to agree consensus on items not reaching consensus and to adapt wording where items were not clear. The patient partners contributed to edits of the paper and are coauthors.
3: Results Outcomes—Report the results of PPI in the study, including both positive and negative outcomes	PPI contributed to the study in several ways, including: - Collating initial evidence - Identifying items for the GRIPP checklist - Considering the evidence and their wider experience—the patients highlighted the importance of including items referring to the context and processes of PPI, suggesting that this affected the impact that PPI had on research - The patient partners, along with other patient organisations and charities, recruited nearly half of all participants for the Delphi survey - The patient partners helped other patients with the technical aspects of completing the online survey, improving the response rate in each Delphi survey round. - The patient partners checked the comprehension of the changed items and comments from the lay perspective between rounds and were integral to helping the researchers keep to the scheduled time of the Delphi survey - Throughout the write-up phase for both the results paper and the methods paper the patient partners contributed to the lay sections and contributed to edits of the paper
4: Discussion Outcomes—Comment on the extent to which PPI influenced the study overall. Describe positive and negative effects	Patient and public involvement in this study was very effective and influenced important aspects of the study, based on the impacts in section 3. This might have been related to several factors. Firstly the patient partners had received training around research methods in previous studies, and were actively involved in a patient and public involvement group attached to the University of Warwick. In addition, the researchers were experienced at involving patient partners in their research. The right processes were in place, as the patient partners were involved from the beginning of the study allowing them to help shape the study from the start allowing them to contribute fully to the study. Having the right context, with a collaborative research team, funding to finance their time, and a supportive attitude of their involvement from EQUATOR and other collaborators, also assisted in the positive impact that PPI had on this study. Pre-existing relationships with patient partners and patients who attended the collaborative consensus event provided a vital context for embedded PPI. However, there were limitations. The methods used to gain consensus had been developed and tested for reliability and validity by EQUATOR in the development of previous guidelines, which limited the possible input from the patient partners in identifying or developing methods to gain consensus on GRIPP2. Furthermore, the time for feedback between Delphi survey rounds was short, and organising times where both researchers and patient partners could meet was difficult. In similar future studies, scheduling of these meetings in advance of the Delphi survey might overcome this limitation.
5: Reflections Critical perspective—Comment critically on the study, reflecting on the things that went well and those that did not, so others can learn from this experience	The PPI in the study was embedded as far as possible into the methods for developing consensus. While not a formal part of EQUATOR methodology, the aim of active collaboration in an attempt to co-produce knowledge worked well. The key challenge was the timescales required to ensure the Delphi survey was completed with appropriate intervals. If this was repeated, these time scales would require extension. We are aware that this process might have limited the extent to which patient partners were able to identify concepts of importance that sit outside of the traditional research paradigm and so may require further development in the future.

^a^An example of using the long form can be obtained from the authors

**Table 4:** Patient and public involvement in GRIPP2 according to BMJ guidance

How was the development of the research question and outcome measures informed by patients’ priorities, experience, and preferences?
Patients were involved in the original systemic review that underpinned GRIPP and actively contributed to identifying the issue of inconsistent reporting, the need for guidance, and the research question.
How did you involve patients in the design of this study?
Patients were involved as research partners in all aspects of the study including identifying the original research question, identifying the need for the original systematic review, and identifying the need for consensus.
Were patients involved in the recruitment to and conduct of the study?
The patient partners, along with other patient organisations and charities, recruited nearly half of all participants for the Delphi survey. They helped pilot the electronic survey for the first phase of the Delphi survey consensus process and helped other patient reps with technical aspects of completing the online survey, hence improving the response rate in each round of the Delphi. They also worked with the researchers to collate comments from each Delphi survey round, to adapt items, and to feed back to the participants for the next Delphi survey round. They checked comprehension of changed items and comments from the lay perspective. The patient partners took part in the consensus workshop, alongside five other patients (*n* = 8/25 in total) to agree consensus on items not reaching consensus and to adapt wording where items were not clear. The patient partners contributed to edits of the paper and are coauthors.
How will the results be disseminated to study participants?
GRIPP2 will be disseminated to all study participants via email. The authors will disseminate via conference presentations. Funding bodies and other journal editors internationally will be encouraged to use GRIPP2.

## Scope and illustration of use

GRIPP2-LF (Table 1) and GRIPP2-SF (Table 2) are the first international, evidence based, community consensus informed guidelines for the reporting of PPI in research. The checklists provide key PPI concepts that authors should report in papers, to enhance the overall quality and transparency of the PPI evidence base. GRIPP2-LF and GRIPP2-SF ultimately aspire to guide PPI reporting in different types of studies, from reporting on PPI in trials (GRIPP2-SF) to reporting of PPI focused studies (GRIPP2-LF). Researchers can use the reporting guideline prospectively to plan PPI in studies and retrospectively as a quality assurance step in the writing up of PPI in publications and reports. Health and social care research funders and research institutions could promote adherence to the GRIPP2 reporting checklist as a means to optimise the creation of transparent, consistent, and high quality PPI evidence. Journal editors could use GRIPP2 reporting checklists to set their reporting expectations for submitted manuscripts. Higher quality reporting will gradually lead to the development of a stronger PPI evidence base that will facilitate more effective synthesis of PPI studies.

GRIPP2 can be used in different ways within a paper. For GRIPP 2-LF the entire paper can be shaped by the guidance, with researchers selecting the items of relevance. With GRIPP2-SF researchers could present all the information in the body of the paper under the relevant reporting titles or in a separate box. Table 3 provides an illustration of GRIPP2 –SF using this study as an example. This table is an illustration of the potential of GRIPP2 reporting. It is purposefully long to demonstrate the type of information it could include. A more specific, shorter form of reporting would also be acceptable, as long as it contained the key information.

## Availability

GRIPP2-SF and GRIPP2-LF are available on the EQUATOR webpage (www.equator-network.org/), or at http://www2.warwick.ac.uk/fac/med/research/hscience/wrn/research/themea.

## Discussion and limitations

GRIPP2-LF and GRIPP2-SF are the first international, evidence based, community consensus informed guidelines for the reporting of patient and public involvement in research. Although consensus was achieved in the development of GRIPP2, further refinements are expected over time as the evidence base underpinning PPI evolves, reflecting the iterative EQUATOR method of guideline development. In addition, it has not yet been possible to conduct any usability testing to understand how GRIPP2 works in practice with different types of study designs. The final consensus meeting did not include international experts because of a restricted budget, which might have limited the discussion from an international perspective. Thus, the next phase of development for GRIPP2-LF and GRIPP2-SF should include wider international application and piloting to test conceptual equivalence in different country contexts. Feedback from researchers using GRIPP2 will help refine it. We have created a comment box on the Warwick Medical School website to facilitate this http://www2.warwick.ac.uk/fac/med/research/hscience/wrn/research/themea.

Guidelines such as the CONSORT statement for randomised controlled trials (RCTs) are regularly updated to reflect changes in health research more widely [19]. Such evolution is particularly important for GRIPP2 because PPI is at a pre-paradigm stage in its development and recognition, reflecting Kuhn’s conceptualisation of how science changes over time with significant paradigm shifts that generate new ways of thinking [20].

While GRIPP2-LF and GRIPP2-SF aim to guide consistent reporting, it is not possible to be prescriptive about the exact content of each item, as the current evidence base is not advanced enough to make this possible [2–4, 21]. Authors should carefully consider the relevance of each GRIPP2 item but recognise that it is sometimes not necessary, or even possible, to include each item in a particular manuscript. With future development of the evidence base, it will be possible to refine GRIPP items, and some may become mandatory.

The success of the PPI in this study may relate to several factors. Firstly, the patient partners had received training around research methods in previous studies and were actively involved in a patient and public involvement group attached to the University of Warwick Medical School. Furthermore, the researchers were experienced at involving patient partners in their research [22]. Finally, good relationships and ways of working were established, which are known as key factors for facilitating high quality PPI [4, 21].

We recognise that GRIPP2 was developed with experts familiar with PPI and that there are still significant challenges in academic culture in enacting the behaviour changes that public involvement requires. PPI needs to become embedded practice within research rather than an option, and both researchers and patients need to recognise their own training and development needs, drawing on the evidence base to guide effective practice.

A further limitation is that GRIPP2-LF and GRIPP2-SF are conceptualised within the culture and language of research. Bearing in mind that the ultimate intention of high quality reporting in PPI is to develop best practice, there is a need to develop a patient or service user version of GRIPP2 to ensure comprehensibility and usefulness and to ensure that patient important concepts indicative of high quality research are included, although these are yet to be identified. This would reflect important changes in academic publishing, where patients are regularly writing and peer reviewing academic papers and require ways of understanding reporting quality [22]. Used alongside other EQUATOR guidance, the intention is to guide the development of a transparent, consistent, and high quality PPI evidence base. More effective synthesis of the PPI evidence base will help to identify best practice, avoid poor practice, and contribute to research that is acceptable, relevant, appropriate, and high quality and that has the potential to generate benefit for all.

## Appendix

Appendix 1: Flow chart of the Delphi Survey. (DOCX 49 kb)
Appendix 2: Consensus meeting outcomes. (DOCX 13kb)
Appendix 3: Results of the Delphi survey for GRIPP2 long form. (DOCX 18 kb)
Appendix 4: Results of the Delphi Survey for GRIPP2 short form. (DOCX 24 kb)
