# Supplementary material for: GRIPP2 reporting checklists: tools to improve reporting of patient and public involvement in research
Source: Res Involv Engagem. 2017 Aug 2;3:13. doi: 10.1186/s40900-017-0062-2 (PMC5611595; doi:10.1186/s40900-017-0062-2)
Supplement: Appendix 1: — Flow chart of the Delphi Survey. (DOCX 49 kb) [file 40900_2017_62_MOESM1_ESM.docx]

**Appendix 1: Flow chart of the Delphi Survey**

**The Consensus Delphi Survey**

Participants asked to rate the GRIPP items on a likert scale from 1= not at all important to 10=very important.

**Delphi Phase 1 (n=143)**

Items reaching consensus (≥8 in Phase 1 and Phase 2), and any additional feedback will be reported to participants.

Participants asked to rate items that have been suggested by respondents in Phase 2.

**Delphi Phase 2 (n=123)**

**Delphi Phase 3 (n=112)**

Items where comments from respondents have suggested that single items contained multiple concepts of differing importance will be delineated. Respondents will be asked to rate each sub item separately.

Participants asked to re-rate the items and respond to the existing comments, if desired.

Participants asked to rate any additional items added from Phase 1 of the Delphi survey

Participants compare their previous rating, and group summary ratings and all anonymised free text.

‘Free text’ comment sections available after each item, for suggestions of refinements, reiterations or additional items.

**Consensus meeting (25 PPI experts)**

Participants asked to rate items requiring a third round of feedback: Those rated of moderate importance (median 6 and 7) after two phases, will be rated again.
