# Supplementary material for: GRIPP2 reporting checklists: tools to improve reporting of patient and public involvement in research
Source: Res Involv Engagem. 2017 Aug 2;3:13. doi: 10.1186/s40900-017-0062-2 (PMC5611595; doi:10.1186/s40900-017-0062-2)
Supplement: Appendix 2: — Consensus meeting outcomes. (DOCX 13kb) [file 40900_2017_62_MOESM2_ESM.docx]

**Appendix 2: Consensus meeting outcomes**

| **Item** | **Outcome of discussion** |
| --- | --- |
| Q2b: Definition: Report how your definition links to those provided by other comparable studies | Q2b: 3 groups agreed to keep the question in and 1 group decided that it could be absorbed into 2a. It was agreed that this item be included but rewording discussed further. |
| Q2c: Concepts and theory development: Report the way in which PPI is being conceptualised | All 4 groups agreed this was similar to 2d and therefore should be absorbed into 2d. |
| Q2d: Concepts and theory development: Report any conceptual or theoretical models, or influences, used in the study | Q2d: All 4 groups agreed this should be included. |
| Methods: economic assessment Q6a: If applicable, report the method used for an economic assessment of PPI | Q6: All 4 groups agreed this item be included. |
| Results: Q7e (ii): If applicable, report any testing of conceptual or theoretical models | Q7e (ii): 3 groups agreed to keep in and 1 group decided that was an overlap with 7 (i) and the word testing was questioned. |
| Discussion: Q8c: Definition: Comment on the definition of PPI used in the study and whether you would suggest any changes | Q8c: 3 groups agreed to keep in and 1 group decided there was an overlap with Q8d. |
