# Supplementary material for: GRIPP2 reporting checklists: tools to improve reporting of patient and public involvement in research
Source: Res Involv Engagem. 2017 Aug 2;3:13. doi: 10.1186/s40900-017-0062-2 (PMC5611595; doi:10.1186/s40900-017-0062-2)
Supplement: Appendix 4: — Results of the Delphi Survey for GRIPP2 short form. (DOCX 24 kb) [file 40900_2017_62_MOESM4_ESM.docx]

**Appendix 4: Results of the Delphi Survey for GRIPP2-SF**

| **Section and Topic** | **Item** | **Round 1**  **Median Scores (IQR)** | **Round 2**  **Median Scores**  **(IQR)** | **Round 3**  **Median Scores**  **(IQR)** |
| --- | --- | --- | --- | --- |
| **1. Aims** | \|  \| **Report the aim of PPI in the study** \| \| --- \| --- \| | **N/A** | **9.0**  **(9.0, 10.0)** | **9.0√**  **(9.0, 10.0)** |
| **2. Methods** | \| **Provide a clear description of methods used for PPI in the study** \| \| \| --- \| --- \| \|  \| | **N/A** | **9.0**  **(9.0, 9.0)** | **9.0√**  **(9.0, 9.0)** |
| **3. Study Results** | **Outcomes: Report the results of PPI in the study, including both positive and negative outcomes** | **N/A** | **9.0**  **(9.0, 10.0)** | **9.0√**  **(9.0, 10.0)** |
| **4. Discussion and conclusions** | **Outcomes: Comment on the extent to which PPI influenced the study overall. Describe positive and negative effects** | **N/A** | **9.0**  **(9.0, 9.0)** | **9.0√**  **(9.0, 9.0)** |
| **5. Critical perspective** | **Comment critically on the PPI in the study, reflecting on the things that went well and those that did not, so others can learn from this experience** | **N/A** | **9.0**  **(9.0, 9.0)** | **9.0√**  **(9.0, 9.0)** |
